# Comparison of Populations Served in Hospital Service Areas With and Without Comprehensive Primary Care Plus Medical Homes

**DOI:** 10.1001/jamanetworkopen.2018.2169

**Published:** 2018-09-28

**Authors:** Taressa K. Fraze, Elliott S. Fisher, Marisa R. Tomaino, Kristen A. Peck, Ellen Meara

**Affiliations:** 1 The Dartmouth Institute for Health Policy and Clinical Practice, Geisel School of Medicine, Dartmouth College, Lebanon, New Hampshire

## Abstract

**Importance:**

Little is known about the types of primary care practices that have chosen to participate in the Comprehensive Primary Care Plus (CPC+) program or about how participation could affect disparities.

**Objective:**

To describe practices that joined the CPC+ model and compare hospital service areas with and without CPC+ practices.

**Design, Setting, and Participants:**

This comparative cross-sectional study identified 2647 CPC+ practices in round 1 (from January 1, 2017; round 1 is ongoing through 2021). Using IMS Health Care Organization Services data, ownership and characteristics of health systems and practices were extracted. Practices participating in the CPC+ program were compared with practices with similar proportions of primary care physicians (>85%) within the 14 regions designated as eligible to participate by the Centers for Medicare & Medicaid Services. Within eligible regions, hospital service areas with (n = 434) and without (n = 322) 1 or more CPC+ practice were compared. Characteristics compared included area-level population demographics (from the US Census Bureau), health system characteristics (from the IMS Health Care Organization Services), and use of health services by Medicare fee-for-service enrollees (Dartmouth Atlas).

**Main Outcomes and Measures:**

Area-level characteristics of all eligible CPC+ regions, areas without a CPC+ practice, and areas with 1 or more CPC+ practices.

**Results:**

Of 756 eligible service areas, 322 had no CPC+ practices and 434 had at least 1 CPC+ practice. Of 2647 CPC+ practices, 579 (21.9%) had 1 physician and 1791 (67.7%) had 2 to 10 physicians. In areas without CPC+ practices, the population had a lower median income ($43 197 [interquartile range, $42 170-$44 224] vs $57 206 [interquartile range, $55 470-$58 941]), higher mean share of households living in poverty (17.8% [95% CI, 17.2%-18.4%] vs 14.4% [95% CI, 13.9%-15.0%]), higher mean educational attainment of high school or less (52.7% [95% CI, 51.7%-53.6%] vs 43.1% [95% CI, 42.1%-44.2%]), higher mean proportion of disabled residents (17.7% [95% CI, 17.3%-18.2%] vs 14.2% [13.8%-14.6%]), higher mean participation in Medicare (21.9% [95% CI, 21.3%-22.4%] vs 18.8% [95% CI, 18.3%-19.1%]) and Medicaid (22.2% [95% CI, 21.5%-22.9%]) vs 18.5% [95% CI, 17.8%-19.2%]), and higher mean proportion of uninsured residents (12.4% [95% CI, 11.9%-12.9%] vs 10.3% [95% CI, 9.9%-10.7%]) (*P* < .001 for all) compared with areas that had a CPC+ practice.

**Conclusions and Relevance:**

According to this study, although a diverse set of practices joined the CPC+ program, practices in areas characterized by patient populations with greater advantage were more likely to join, which may affect access to advanced primary care medical home models such as CPC+, by vulnerable populations.

## Key Points

**Question:**

What type of primary care practices join the Comprehensive Primary Care Plus program, and which patient populations do those practices serve?

**Findings:**

This comparative cross-sectional study of 756 areas eligible for the Comprehensive Primary Care Plus program found that a diverse set of primary care practices joined the program. Practices located in wealthier areas with higher education levels and less use of inpatient services were more likely to join.

**Meaning:**

Voluntary advanced primary care medical home models, such as the Comprehensive Primary Care Plus program, may leave behind practices serving vulnerable populations.

## Teaser

This comparative cross-sectional study describes the practices of the Comprehensive Primary Care Plus medical home program model and compares hospital service areas with and without these practices using data from the US Centers for Medicare & Medicaid Services.

## Introduction

With the aim of improving the quality, accessibility, and efficiency of care, the Comprehensive Primary Care Plus (CPC+) model is the largest test by the Centers for Medicare & Medicaid Services (CMS) of an advanced primary care medical home model.^1^ The CPC+ is a voluntary multipayer model that combines primary care redesign with efforts to restructure payment through prospective care management payments and performance-based incentives for care improvement. Although such models have the potential to enhance care for the nation’s most vulnerable patients, evidence suggests that care delivery reform models may widen gaps across communities, because physicians in areas with low resources may choose not to participate.^2,3,4,5,6^ Little is known about the types of practices participating in medical home models or whether participation may affect disparities. In this study, we describe the inaugural CPC+ practices and compare hospital service areas with and without a CPC+ practice.

## Methods

This study followed the Strengthening the Reporting of Observational Studies in Epidemiology (STROBE) reporting guideline.^7^ To examine the characteristics of practices participating in the CPC+ program, we used publicly available data from the CMS^8^ and identified 2647 CPC+ practices in round 1 (93.0%) from January 1, 2017 (round 1 is ongoing through 2021). We extracted data describing ownership and characteristics of health systems and practices using IMS Health Care Organization Services (HCOS) data from 2016. The HCOS data included the American Medical Association Masterfile data to identify physicians in practices. The CPC+ practices were first identified in the HCOS by matching the names of business and physical addresses. We then conducted web searches on the remaining CPC+ practices to identify physicians in those practices or alternative practice names to link with HCOS data. Dartmouth College’s Committee on the Protection of Human Subjects approved this study and did not require informed consent for the use of publicly available databases.

We compared CPC+ practices with other primary care practices operating within the 14 eligible regions that the CMS designated for participation. The CPC+ program operates statewide in 11 states and in select counties in 3 additional states. Comparison practices included at least 1 primary care physician (practices with only specialist physicians were excluded), and we required a similar proportion of primary care physicians (>85%) as observed in the CPC+ practices. We included family medicine, geriatric medicine, internal medicine, and general medicine specialists as primary care physicians (pediatricians were not included as primary care physicians).

Within eligible regions, we compared hospital service areas with and without 1 or more CPC+ practice. We examined area-level population demographics (US Census Bureau),^9^ health system characteristics (IMS HCOS), and use of health services of Medicare fee-for-service enrollees (Dartmouth Atlas).^10^ We compared area-level characteristics of all eligible CPC+ areas, areas without a CPC+ practice, and areas with 1 or more CPC+ practice.

### Measures

Measures characterizing primary care practices and physicians were based on 2016 IMS HCOS data and the American Medical Association’s Masterfile for physician-level counts. We used the CMS 2017 provider of services files to identify the presence of federally qualified health centers and rural health clinics. Data from 2014 100% Medicare fee-for-service claims were obtained from the Dartmouth Atlas, which adjusted measures of use of services for age, sex, and race/ethnicity in the Medicare fee-for-service population. Dartmouth Atlas measures included number of Medicare enrollees, dual eligibility status, and measures of use of services. Hospital service area characteristics and insurance coverage (except dual eligibility) were based on 2016 US Census Bureau 5-year population estimates from the American Community Survey (2012-2016). Hospital service areas are collections of zip codes, and census information was aggregated to the hospital service area level as a population-weighted sum of the Census Bureau’s value from each 2016 zip code tabulation area. The weights were calculated as the proportion of the total hospital service area population, which was summed from the Census Bureaus’ 2016 zip code tabulation area total population.

Urbanicity categories align with the US Census Bureau’s definitions of urban areas, which are based on the number of people living in the zip code tabulation area plus other nonpopulation criteria. Urban refers to census urbanized areas (≥50 000 people); suburban, census urban clusters (2500 to <50 000 people). The rural category has a population of fewer than 2500 people. The proportion of zip code tabulation area population in each urbanicity category comes from the 2010 decennial census, which was then weighted to the 2016 population data.

### Statistical Analysis

We compared characteristics of CPC+ practices with other practices in eligible CPC+ regions using descriptive statistics with means for continuous variables and proportions for categorical variables (Table 1). To compare hospital services areas with and without CPC+ practices, we report descriptive statistics using means with 95% CIs and medians with interquartile ranges (IQRs) (Table 2). Measures of use of health services from the Dartmouth Atlas were adjusted by age, sex, and race/ethnicity; the remaining variables were not adjusted. We used 2-tailed *t* tests or Wilcoxon rank sum tests, as appropriate, to calculate significance. Significance was set at *P* < .05, but most differences were significant at *P* < .001, as shown in Table 2. Rates of missing data were low except for the measure of annual 30-day hospital readmissions.

**Table 1.** Characteristics of Primary Care Practices Participating in CPC+ Compared With Other Practices in CPC+-Eligible Regions

Characteristic	Primary Care Practices	
	Regions Eligible for CPC+ Participation (n = 12 020)	CPC+ Practices (n = 2647)
No. of primary care physicians, mean (SD)	1.7 (1.9)	3.7 (3.9)
No. of specialist physicians, mean (SD)	0.02 (0.2)	1.3 (4.9)
Proportion of primary care physicians, mean (SD), %	99.9 (0.01)	87.1 (0.3)
Practices with only primary care physicians, No. (%)	11 863 (98.7)	1872 (70.7)
Practices with nurse practitioners, physician assistants, or clinical nurse specialists, No. (%)	3458 (28.8)	1338 (50.5)
Practice size, No. (%) of physicians		
1 (solo)	8619 (71.7)	579 (21.9)
2-10 (small)	3308 (27.5)	1791 (67.7)
11-20 (medium)	71 (0.6)	172 (6.5)
≥21 (large)	22 (0.2)	77 (2.9)
System		
Practices in a system, No. (%)	2127 (17.7)	1549 (58.5)
System-based practices in a system with a hospital, No. (%)	1530 (71.9)	1177 (76.0)

Abbreviation: CPC+, Comprehensive Primary Care Plus.

**Table 2.** Characteristics of Hospital Services Areas With and Without CPC+ Practices

Characteristic	Hospital Service Area			*P* Value^a^
	Eligible for CPC+ Program (n = 756)	Without CPC+ Practices (n = 322)	With CPC+ Practices (n = 434)	
Total population, median (IQR)	41 237 (16 635-96 653)	17 319 (8405-35 612)	75 618 (34 369-141 793)	<.001
Residents in urban census tracts, median (IQR), %	0.0 (0.0-73.6)	0.0 (0.0-0.0)	58.6 (0.0-89.9)	<.001
Residents in suburban census tracts, mean (95% CI), %	24.8 (23.0-26.5)	30.5 (27.7-33.2)	20.5 (18.2-22.8)	<.001
Residents in rural census tracts, mean (95% CI), %	44.6 (42.5-46.8)	60.6 (57.6-63.7)	32.8 (30.4-35.2)	<.001
Household income, median (IQR), $	51 239 (50 046-52 433)	43 197 (42 170-44 224)	57 206 (55 470-58 941)	<.001
Residents below the poverty level, mean (95% CI), %	15.9 (15.4-16.3)	17.8 (17.2-18.4)	14.4 (13.9-15.0)	<.001
Residents disabled, mean (95% CI), %	15.7 (15.4-16.0)	17.7 (17.3-18.2)	14.2 (13.8-14.6)	<.001
Residents with high school educational attainment or less, mean (95% CI), %	47.2 (46.4-48.0)	52.7 (51.7-53.6)	43.1 (42.1-44.2)	<.001
No. of nonwhite residents, median (IQR)	15.1 (8.3-28.8)	12.8 (6.8-26.5)	17.8 (9.7-30.9)	<.001
Residents 18 y and younger, mean (95% CI), %	22.4 (22.2-22.6)	22.3 (21.9-22.7)	22.4 (22.2-22.7)	.53
Residents 75 y and older, mean (95% CI), %	7.4 (7.3-7.6)	8.0 (7.8-8.3)	7.0 (6.8-7.2)	<.001
Age, median (IQR), y	41.0 (40.6-41.3)	41.8 (41.3-42.4)	40.3 (41.3-42.4)	<.001
Health insurance coverage, mean (95% CI), %				
Medicare	20.1 (19.7-20.4)	21.9 (21.3-22.4)	18.8 (18.3-19.1)	<.001
Medicaid	20.1 (19.6-20.6)	22.2 (21.5-22.9)	18.5 (17.8-19.2)	<.001
Employer	52.6 (51.9-53.4)	47.9 (46.9-48.9)	56.2 (55.1-57.2)	<.001
Uninsured	11.2 (10.9-11.5)	12.4 (11.9-12.9)	10.3 (9.9-10.7)	<.001
Eligible for Medicare and Medicaid	20.6 (20.1-21.1)	22.6 (21.8-23.3)	19.1 (18.5-19.7)	<.001
Health system characteristics				
No. of specialist physicians per 100 000 population, median (IQR)	97.2 (49.5-159.5)	56.3 (26.4-102.43)	131.7 (80.8-198.11)	<.001
No. of primary care physicians per 100 000 population, median (IQR)	62.7 (46.2-83.2)	55.7 (41.5-79.4)	66.4 (50.4-85.5)	<.001
No. of nurse practitioners, physician assistants, and clinical nurse specialists per 100 000 population, median (IQR)	61.9 (39.2-90.0)	65.0 (42.1-94.1)	60.4 (37.2-86.8)	.09
Practices in system, median (IQR), %	6.0 (2.0-16.0)	4.0 (1.0-5.0)	12.0 (5.0-25.0)	<.001
Includes a federally qualified health center, mean (95% CI), %	64.6 (61.1- 68.0)	52.2 (46.7-57.7)	73.7 (69.6-77.9)	<.001
Includes a rural health clinic, mean (95% CI), %	60.1 (56.6-63.6)	74.5 (69.8-79.3)	49.3 (44.6-54.0)	<.001
Medicare fee-for-service enrollees				
No. of Medicare enrollees, median (IQR)	4537 (2030-10 244)	2134 (1223-4113)	7893 (4102-14 943)	<.001
No. of reimbursements per enrollee, mean (95% CI)	9616 (9515-9716)	9667 (9499-9835)	9577 (9455-9700)	.39
Annual deaths, mean (95% CI), %	4.7 (4.6-4.7)	4.9 (4.8-5.0)	4.5 (4.4-4.6)	<.001
Annual 30-d readmission, mean (95% CI), %	14.8 (14.7-15.0)	15.1 (14.8-15.4)	14.7 (14.5-14.9)	.01
Ambulatory care sensitive discharges per 1000 enrollees, mean (95% CI), %	59.9 (58.3-61.6)	68.4 (65.4-71.5)	53.9 (52.2-55.6)	<.001
Hospital discharges per 1000 enrollees, mean (95% CI), %	272.9 (268.8-277.0)	281.6 (274.9-288.4)	266.4 (261.5-271.4)	<.001

Abbreviations: CPC+, Comprehensive Primary Care Plus; IQR, interquartile range.

^a^ Calculated using 2-tailed *t* tests or Wilcoxon rank sum tests, as appropriate.

## Results

Of the 2647 CPC+ practices, 1791 (67.7%) had 2 to 10 physicians (small) and 579 (21%) had 1 physician, whereas the 12 010 comparison practices most commonly were solo physicians (8619 [71.7%]). One thousand three hundred thirty-eight CPC+ practices (50.5%) included nonphysician clinicians such as nurse practitioners, physician assistants, or clinical nurse specialists, whereas only 3458 comparison practices (28.8%) included nonphysician clinicians. One thousand five hundred forty-nine CPC+ practices (58.5%) were owned by a health care system compared with 2127 comparison practices (17.7%).

In qualifying CPC+ regions, we identified 756 hospital service areas, of which 322 (42.6%) did not have a CPC+ practice, 113 (14.9%) had 1 CPC+ practice, and 321 (42.5%) had 2 or more CPC+ practices. A mean of 6.5 CPC+ practices (95% CI, 5.6-7.5) were in hospital service areas that included a CPC+ practice. Although CPC+ practices were present in areas that were urban, suburban, and rural, the urban, populous areas more often included CPC+ practices (median population for areas with CPC+ practices, 75 618 [IQR, 34 369-141 793] vs 17 319 [IQR, 8405-35 612] for areas without CPC+ practices). Compared with areas with a CPC+ practice, areas without CPC+ practices were characterized by disadvantages, including a lower median income ($43 197 [IQR, $42 170-$44 224] vs $57 206 [IQR, $55 470-$58 941]), higher mean share of households living in poverty (17.8% [95% CI, 17.2%-18.4%] vs 14.4% [95% CI, 13.9%-15.0%]), higher mean educational attainment of high school or less (52.7% [95% CI, 51.7%-53.6%] vs 43.1% [95% CI, 42.1%-44.2%]), higher mean proportion of disabled residents (17.7% [95% CI, 17.3%-18.2%] vs 14.2% [13.8%-14.6%]), higher mean participation in Medicare (21.9% [95% CI, 21.3%-22.4%] vs 18.8% [95% CI, 18.3%-19.1%]) and Medicaid (22.2% [95% CI, 21.5%-22.9%]) vs 18.5% [95% CI, 17.8%-19.2%]), and higher mean proportion of uninsured residents (12.4% [95% CI, 11.9%-12.9%] vs 10.3% [95% CI, 9.9%-10.7%]) (*P* < .001 for all). Medicare fee-for-service enrollees in areas without a CPC+ practice had significantly higher use of inpatient services overall (mean per 1000 enrollees, 281.6 [95% CI, 274.9-288.4] vs 266.4 [95% CI, 261.5-271.4]) and potentially avoidable discharges (mean per 1000 enrollees, 68.4 [95% CI, 65.4-71.5] vs 53.9 [95% CI, 52.2-55.6]) (*P* < .001) than enrollees in areas with a CPC+ practice (Table 2).

## Discussion

The CPC+ model attracted small practices, and most were owned by a health system. The CPC+ program aimed to recruit a diverse set of practices in terms of size, ownership, and composition.^1^ Primary care practices located in areas with higher income and educational levels and lower use of inpatient services were more likely to join the CPC+ model compared with practices in other areas. Practices located in areas with more health care resources per capita were also more likely to join the CPC+ program. Although the goal of the CPC+ program is to give practices financial resources to make more flexible investments to improve quality of care, low participation in less advantaged areas suggests that practices most in need of additional resources may not access them.

Several features of the CPC+ model could contribute to this misalignment of the program with needs. First, clinics that predominately serve vulnerable populations, such as federally qualified health centers and rural health clinics, are excluded from many reform efforts, including the CPC+ program.^8^ These clinics and the patients they serve may benefit from care delivery and payment reform efforts. We found that 74.5% (95% CI, 69.8%-79.3%) of areas without CPC+ practices included a rural health clinic that could, by design of the CPC+ program, influence which areas and patients are able to access advanced care delivery efforts. Complex billing and payment models for federally qualified health centers and rural health clinics create challenges to participation in programs like the CPC+ model. Structural barriers, such as payer composition and participation, could also affect participation in the program in areas without CPC+ practices.

A more robust approach to attract practices in vulnerable areas requires understanding why practices did not join. Because disadvantaged areas have lower resources, they may face special challenges meeting performance targets, which rely, in part, on absolute performance thresholds.^11,12^ For similar reasons, these practices may have difficulty meeting requirements around all 5 key functions required of CPC+ participants: access, care management, comprehensiveness and coordination, patient and caregiver engagement, and planned care and population health.^1^ These care delivery requirements may be especially challenging for practices that serve vulnerable populations because they may have lower capacity and fewer resources to invest in the required intensive transformation activities.^13,14^

### Limitations

Our study has several key limitations. Secondary data sources, such as IMS HCOS data on primary care practices, could include errors because practice characteristics can change regularly. Our use of aggregate Medicare measures required us to omit some of the CMS requirements for CPC+ participation, such as the minimum number of Medicare patients attributed to a practice, which could affect comparison practices. Future analyses could replicate the attribution methods of the CMS and compare patient composition of practices. Despite these limitations, our study offers valuable early insights into the types of practices that choose to join voluntary advanced primary care medical homes and highlights some implications for policymakers to consider.

## Conclusions

The CPC+ model, created to improve care delivery, may leave behind patients in communities with the greatest needs. The CMS and other participating payers may need to modify program requirements to encourage practices that serve more disadvantaged areas to join.
